# *Aspicularis tetraptera* Induced Hematological Parameters in Infected and Vaccinated Mice

**Published:** 2012

**Authors:** S Gaherwal, S Solanki, M M Prakash, N Wast

**Affiliations:** Department of Biotechnology, Gov. Holkar Science College, Indore (M.P.), India

**Keywords:** *Aspiculuris tetraptera*, RBC, Hb, Serum protein, Nematode Haematological parameters, Immune response

## Abstract

**Background:**

The present study deals with the effect of helminthic infection as Nematode parasite like *Aspiculuris tetraptera* on the haematological parameters of infected and vaccinated mice.

**Methods:**

Totally 15 mice were used. Five mice were used for positive control, 5 mice used for negative control and 5 mice used for experiment. The hematological parameters were studied viz. RBC, Hb, and serum protein values.

**Results:**

The mice carrying heavy infection showed decrease in the Hb, RBC, and serum protein but in the vaccinated mice, all studied parameters were become on normal range. The level of immune response was assessed based on above studied hematological parameters in infected and vaccinated mice with *Aspiculuris tetraptera*.

**Conclusion:**

The increased value of RBC, Hb and Serum protein in infected and vaccinated mice compared to infected and non vaccinated suggested the involvement of blood parameters in immune response. This study also proves that somatic antigen of *A. tetraptera* was effective in imparting immunity in mice

## Introduction

Chronic intestinal nematode infections pose a significance threat to the health of man and domestic animals. The mechanisms employed to evade host immunity are still poorly understood but there is growing consensus that a number of diver's species exert an immunomodulatory influence, causing potentially host-protective effecter responses to be down-regulated (1–4), although most of these studies have been carried out on nematodes. *A. tetraptera* nematodes also provide a good model for studying the mechanisms of innate and acquired immunity to nematode infection.

The *Aspiculuris tetraptera* - mouse pinworm is useful parasite model of the human parasite *Enterobius vermicularis*. Behnke reported the immune expulsion of the nematode *Aspiculuris tetraptera* from mice given primary and challenge infections (5). Some scientist reported the analysis of somatic antigens extracted from *Aspiculuris tetraptera* (Oxyuridae) and their role in eliciting immune response in laboratory mice (6).

The expulsion of some nematode parasites from the host during the second and third weeks of a primary infection is well documented in the literature (7–11) and it is invariably accompanied by an acquired immunity to further infection manifest by a more rapid expulsion of challenge infection, and a smaller residual worm burden persisting after the events of immunity (12, 13). In order to ascertain whether the expulsion of *A. tetraptera* was mediated by immunological phenomena, it was important to know whether a primary infection initiated a state of acquired immunity. Therefore, the present study was under taken to find out the role of blood cells in active immunization against *A. tetraptera* in mice.

## Materials and Methods

### Experimental Animal

The Inbred female Swiss albino mice, *Mus musculus albinus* of 6-8 weeks old and 15-20 gm in weight were selected as an experimental animals. Totally 15 mice were used. Five mice were used for positive control, 5 mice used for negative control and 5 mice used for experiment

.

### Experimental Parasite

For the present investigation *A. tetraptera* was selected as an experimental parasite and it being routinely maintained in the laboratory by serial passage

### Preparation of Somatic Antigen

Somatic antigens were prepared by homogenization and lyophilization. The eggs, the larvae and the adult stages of the worms, were washed thoroughly and homogenized separately in the protein free culture medium (Hopkins serum free culture medium). The homogenate were lyophilized and kept at 4 °C.

### Immunization of Mice

An initial dose of 0.4 ml of the suspension with 0.2 ml of antigenic sample containing the required content Protein content of antigenic sample was estimated by method of Lowry (14) and 0.2 ml of Freund's complete adjuvant (FCA) was injected subcutaneously (SC) for immunization. The protein content of the antigenic sample varied according to the experiments, however, the booster dose was of 0.2 ml, containing required amount of the of protein without FCA.

### Preparation of inoculums for infection

The 100 viable eggs were fed to each mouse. After inoculation, mice were kept in cages, labelled according to the design of experiments, were fed routinely with the same standard diet.

### Collection of the blood samples, separation of serum

Blood from experimental and control mice was collected by cardiac puncture under mild ether anesthesia, before incision each mouse were swabbed with 90% alcohol, heart exposed, blood collected from the ventricle by a 2 ml sterilized dry glass syringe fitted with a suitable in cold overnight for clotting after which serum carefully pipetted out in to clean sterilized serum collecting tubes and stored at -20^O^C until required

### Haemoglobin analysis and RBC counting

Blood from each experimental and control groups of mice was collected from the hearts with a sterile disposable syringe. Haemoglobin percentage analysis and RBC counting were done by Schalm method (15).

### Analysis of serum protein

Blood from each experimental and control groups of mice was collected from the hearts with a sterile disposable syringe, allowed to clot at room temperature for 1 h and then centrifuged to separate serum, which was then used for the estimations of total protein, albumin and globulins by the Biuret and dye methods (16).

## Results

### RBC and Hb value

Results of RBC count in mice infected and vaccinated with somatic antigens are summarized in the tables (1). The RBC count in NINVC_1_ group was found 9.22 mm^3^. In INVC_2_ group, the value decreased to 7.6 mm^3^ on 21^st^ day p.i. When the experimental mice vaccinated with adult somatic antigen the values increased to 11.6 mm^3^ on 21^st^ day p.i. as compared to control-2.

Results of Hb% in mice infected and vaccinated with somatic antigens are summarized in the tables (2). The hemoglobin values in NINVC_1_ group were found 14.6 mg/100ml. In INVC_2_ group, the value decreased to 11.6 mg/100ml on 21^st^ day p.i. When the experimental mice vaccinated with adult somatic antigen the value increased 15 mg/100ml on 21^st^ day post infection as compared to those of controls. All the values obtained in experimental group were statistically significant when compared to those of the controls.

### Serum protein changes

Results of serum protein changes in mice infected and vaccinated with somatic antigens are summarized in the Tables 1–3. In non-infected non-vaccinated control-1, albumin value was 59.28 and globulin value 40.72. In infected non-vaccinated control-2, albumin value was 31.19 and globulin value 68.81 on 21^st^ day post infection. All the values obtained in experimental group were statistically significant when compared to those of the controls. Whereas, in experimental group vaccinated with somatic antigen serum protein values were changed. In the infected vaccinated group, albumin, and globulin value increased as compared to infected non-vaccinated group and similar to non-infected and non- vaccinated group.

**Table 1:** R.B.C. Counts of taken on 21^st^ day post infection from mice infected with *A. tetraptera* and vaccinated with somatic antigen of adult worm

Group	Group name	R.B.C. (10^6^/mm^3^)
1	NINVC_1_	9.22±0.1228
2	INVC_2_	7.6±0.160
3	IVASoAg	11.60±0.49

**Table 2:** Hemoglobin values of taken on 21^st^ day post infection from mice infected with *A. tetraptera* and vaccinated with somatic antigen of adult worm

Group	Group name	Hb% (mg/100ml)
1	NINVC_1_	14.6±0.980
2	INVC_2_	11.6±0.375
3	IVASoAg	15.0±0.02

**Table 3:** Serum Protein Analysis value of taken on 21^st^ day post infection from mice infected with *A. tetraptera* and vaccinated with somatic antigen of adult worm

Group No.	Groups Name	Serum protein changes	
		Albumin	Globulin
1	NINVC_1_	59.28±0.757	40.72±0.247
2	INVC_2_	31.19±0.052	68.81±0.257
3	IVASoAg	61.24±0.831	38.76±0.441

NINVC_1_: Non infected Non vaccinated control – 1

INVC_2_:Infected Non vaccinated control – 2

IVASoAg:Infected vaccinated with adult worm somatic antigen.

## Discussion

In the present investigation, RBC counts and hemoglobin value decreased in the *A*. *tetraptera* infected mice. When the mice vaccinated with adult somatic antigen, RBC count and hemoglobin value increased. RBC counts and hemoglobin value were decreased in infected mice. This may be related to the bleeding due to injuries. Although, increased RBC counts and hemoglobin values due to the killing expulsion of *A. tetraptera* from gastrointestinal track in vaccinated mice.

The present study revealed a marked reduction in hemoglobin and RBC count, which confirmed the observation of early workers (17, 18) who observed decreased values of hemoglobin and RBC counts in lambs in relation to nematode infection. The reduced RBC counts and Hb values infected group may be attributed to the bleeding due to injuries caused by parasites similar to that described by many scientists (19, 20). Increase the RBC counts and hemoglobin in vaccinated mice is due the killing and expulsion of parasite from gastrointestinal tract (21, 22).

Helminthic infections cause a decrease in albumin and increase in globulin. Animals infected with helminth parasites have also shown marked changes in their serum protein fractions. A significance changes in serum protein may be due to the depletion in the level of albumin. Although the level of globulin increases, the extent of depletion in albumin could not have been compensated for, and consequently the level of total protein fell. Depletion of total protein content of serum may also be due to the breakdown of protein under parasitic influence (23, 24).

**Albumin:** Albumin level decreased in infected mice, because albumin is known to serve as an important nutrient for the developing worms. Comparative increase albumin level in vaccinated mice may be attributed to the killing or expulsion of the worm by the immunogens (23, 24).

**Globulin:** Globulin level increased in vaccinated mice, comparative to infected non-vaccinated control mice, because globulin containing antibodies. Increased values of globulins in the experimental groups indicate stimulation of production of antibodies; and these antibodies are responsible for manifestation of host immunity against parasitic infection (23, 24).

Nematode infection has been observed (18, 25) to cause a decrease in albumin and increase in globulin mainly due a rise in the antibodies. Of particular interest is the pronounced increase in the mean serum globulin levels observed in experimental groups, which clearly showed the efficiency of the immune mechanism during the nematode infection, as it is known that IgG1, IgG2 and IgE classes of immunoglobulins are responsible for conferring protective immunity (26–28). The results of the present investigation (serum protein changes) are supported by above-mentioned authors.

In conclusion, the increased value of RBC, Hb and serum protein in infected and vaccinated mice compared to infected and non vaccinated suggested the involvement of blood parameters in immune response. This study also proves that somatic antigen of *A. tetraptera* was effective in imparting immunity in mice.
